# Simultaneous real-time PCR detection of nine prevalent sexually transmitted infections using a predesigned double-quenched TaqMan probe panel

**DOI:** 10.1371/journal.pone.0282439

**Published:** 2023-03-06

**Authors:** Ha T. V. Bui, Huyen T. Bui, Son V. Chu, Huyen T. Nguyen, Anh T. V. Nguyen, Phuong T. Truong, Thang T. H. Dang, Anh T. V. Nguyen

**Affiliations:** 1 Key Laboratory of Enzyme and Protein Technology, VNU University of Science, Vietnam National University, Hanoi, Vietnam; 2 ANABIO R&D Ltd, Hanoi, Vietnam; 3 Department of Microbiology, Bach Mai Hospital, Hanoi, Vietnam; 4 Department of Microbiology, Hanoi Obstetrics & Gynecology Hospital, Hanoi, Vietnam; Rutgers Biomedical and Health Sciences, UNITED STATES

## Abstract

Sexually transmitted diseases are major causes of infertility, ectopic pregnancy, and premature birth. Here, we developed a new multiplex real-time polymerase chain reaction (PCR) assay for the simultaneous detection of nine major sexually transmitted infections (STIs) found in Vietnamese women, including *Chlamydia trachomatis*, *Neisseria gonorrhoeae*, *Gardnerella vaginalis*, *Trichomonas vaginalis*, *Candida albicans*, *Mycoplasma hominis*, *Mycoplasma genitalium*, and human alphaherpesviruses 1 and 2. A panel containing three tubes × three pathogens/tube was predesigned based on double-quenched TaqMan probes to increase detection sensitivity. There was no cross-reactivity among the nine STIs and other non-targeted microorganisms. Depending on each pathogen, the agreement with commercial kits, sensitivity, specificity, repeatability and reproducibility coefficient of variation (CV), and limit of detection of the developed real-time PCR assay were 99.0%–100%, 92.9%–100%, 100%, <3%, and 8–58 copies/reaction, respectively. One assay cost only 2.34 USD. Application of the assay for the detection of the nine STIs in 535 vaginal swab samples collected from women in Vietnam yielded 532 positive cases (99.44%). Among the positive samples, 37.76% had one pathogen, with *G*. *vaginalis* (33.83%) as the most prevalent; 46.36% had two pathogens, with *G*. *vaginalis* + *C*. *albicans* as the most prevalent combination (38.13%); and 11.78%, 2.99%, and 0.56% had three, four, and five pathogens, respectively. In conclusion, the developed assay represents a sensitive and cost-effective molecular diagnostic tool for the detection of major STIs in Vietnam and is a model for the development of panel detections of common STIs in other countries.

## Introduction

Sexually transmitted diseases (STDs) are a public health burden worldwide. STDs can cause severe fetal and neonatal mortality, ectopic pregnancy, infertility, and genital neoplasia. According to the World Health Organization, more than 1 million sexually transmitted infections (STIs) are acquired every day worldwide, with an estimated 374 million new infections each year [1]. Many STIs are generally asymptomatic in the early stages of infection, which increases the potential for undetected transmission. STIs are caused by various microorganisms such as bacteria, viruses, yeast, and protozoa. Common STIs include, but are not limited to, *Chlamydia trachomatis* (CT), *Neisseria gonorrhoeae* (NG), *Candida albicans* (CA), *Mycoplasma genitalium* (MG), *Trichomonas vaginalis* (TV), *Mycoplasma hominis* (MH), *Gardnerella vaginalis* (GV), and human alphaherpesvirus (HSV) types 1 (HSV-1) and 2 (HSV-2). In Vietnam, the prevalence of these nine STIs is relatively high as indicated by the following studies [2–5]: (i) the rates of 6.0% CT, 0.13% NG, and 0.8% TV detected in 800 pregnant women in Hanoi, Vietnam, were the highest [3]; (ii) the highest prevalence of HSV-2 (4%), NG (34%), and CT (19%) have been found in 205 men who have sex with men [4]; and (iii) the four most prevalent causal pathogens found in heterosexual men are CT, NG, MG, and *Ureaplasma* species, with a total prevalence of 40.7% [5]. Unfortunately, STIs present similar clinical symptoms, making the clinical differentiation of pathogens very difficult. Therefore, it is critical to develop a sensitive, multitarget, and high-throughput approach for detecting common STIs. Furthermore, rapid and accurate diagnosis is essential to prevent further transmission, provide urgent intervention, implement early treatment, and lower patient care expenses. Cell culture, microscopic inspection, enzyme immunoassay, and other serological techniques are common STIs diagnostic algorithms; however, they have certain limitations in terms of sensitivity and a long turnaround time, especially for detecting HSV types 1 and 2 and bacterial *Mycoplasma* species owing to difficulties in culturing [6, 7].

Molecular tests, such as real-time polymerase chain reaction (PCR) and hybridization-based mini-array, are more advantageous because they offer shorter turnaround time than the conventional methods [8–15]. Representative commercially available kits for multiplex real-time PCR detection of common STIs include (i) kits for the detection of 2–3 STIs such as Abbott m2000 RealTime CT/NG (Abbott Molecular Inc., Des Plaines, IL, USA) [16], Cobas Amplicor CT/NG test (Roche Molecular Diagnostics, Branchburg, NJ, USA) [17, 18], Aptima Combo 2 Assay for CT/NG (Hologic/Gen-Probe, San Diego, CA, USA) [19], BD ProbeTec CT/GC Q^x^ Amplified DNA assay (Becton Dickinson, Sparks, MD, USA) [20], Xpert CT/NG Assay (Cepheid, Sunnyvale, CA, USA) [21], and Certest VIASURE *C*. *albicans*, *G*. *vaginalis*, and *T*. *vaginalis* Real Time PCR Detection Kit (CerTest Biotec S.L., Zaragoza, Spain) [22]; and (ii) kits for detection of 7–9 STIs, such as Allplex STI Essential Assay Q (MH, UU) (Seegene, Seoul, Republic of Korea) including CT/MG/MH/NG/TV, *Ureaplasma parvum* (UP), *U*. *urealyticum* (UU) [23] or FTD STD9 kit (Fast Track Diagnostics, Junglinster, Luxembourg) including CT/NG/MG/TV/UU/UP/GV/HSV-1/HSV-2 [24]. Although a number of commercial multiplex real-time PCR kits have been developed so far, they are expensive (about 12–15 USD/test for nine STIs, as import cost into Vietnam) and do not cover the major nine STIs found in Vietnam, which makes them unsuitable for screening purposes in hospitals and centers for disease control (CDCs) in developing countries.

Therefore, this study focused on the development of a sensitive simultaneous real-time PCR assay utilizing double-quenched TaqMan probes for the detection of nine STI pathogens commonly found in Vietnam and may also be found in other countries. The advantage of double-quenched TaqMan probes is the inclusion of a second internal quencher (named as ZEN or TAO) in the probes that shortens the distance between the 5′ donor dye and quencher and, in concert with the 3′ quencher, provides greater overall dye quenching, lowers background, and increases signal detection in real-time PCR experiments. This “in-house” real-time PCR assay can be regarded as a model for the development of other panels suited for screening prevalent STIs in countries where the economic and epidemiological status does not allow for large-scale usage of imported kits.

## Materials and methods

### Ethical issues

An approval from the medical ethics council IRB-1906 was received on April 28, 2020, at the Dinh Tien Hoang Institute of Medicine, and the study was conducted from December 2020 to July 2022. Collection of patients’ vaginal swab samples from patients visiting hospitals for vaginal examination was performed in accordance with research ethics regulations in medicine. Patients have signed informed written consent forms (S1 File) to participate in the study after they were provided with sufficient information on the research and confidentiality of personal information. All participants’ information was kept private. Medical records were reviewed to gather relevant research information, and the research-related reports did not contain the participants’ names or any other identifying information. The inclusion criteria were as follows: women, aged 18–66 years, living in Northern Vietnam, and clinical symptoms typical of vaginal infections, with a high degree of inflammation, as indicated by high leukocyte densities.

### Collection of standards and vaginal swab samples, and DNA extraction

First, a total of thirty-nine standard microbial strains and DNA controls were collected to analyze the cross-reactivity of the developed assay. These included microbial standard strains (*n* = 10) obtained from the American Type Culture Collection (ATCC) and Vietnam Type Culture Collection (VTCC), DNA controls (*n* = 23) provided as gifts from the collections at the Departments of Microbiology at Bach Mai Hospitals and Hanoi Obstetrics & Gynecology Hospital (Hanoi, Vietnam), and DNA controls (*n* = 6) obtained from Vircell Microbiologists (Granada, Spain).

Second, coded reference vaginal swab samples (*n* = 136) that were tested positive or negative for the nine STIs using commercial CE-IVD real-time PCR kits were provided by research teams at Bach Mai Hospital, National Hospital of Dermatology and Venereology, and Bac Ninh Center for Disease Control (CDC). The coding of reference samples was blind to most investigators and technicians (except the PI, data analyst, and team leaders at the supplying division). These reference samples were used for analyzing agreement with commercial kits, specificity, and sensitivity of the developed assay.

Finally, random gynecological samples (including original genitourinary secretion cotton swabs and processed swab samples, *n* = 535) from patients who underwent gynecological examinations were collected and preserved according to routine procedures at Bach Mai and Hanoi Obstetrics & Gynecology Hospitals between December 2020 and July 2022. They were used to create the profile of STI prevalence in Vietnamese women using the developed assay.

All microbial standard strains and vaginal specimens were pretreated with 1 mL of 0.9% physiological saline solution and vortexed vigorously for 30 s. Nucleic acids (DNA/RNA) from 200 μL specimens were extracted using the QIAamp DNA Mini Kit (Qiagen, Hilden, Germany), according to the manufacturer’s instructions. Nucleic acid samples were eluted with 100 μL elution buffer and stored at -80°C until further use.

### Design of primer, probe, and positive control panel and performance for real-time PCR assays

Primers and probes were designed and modified to amplify specific sequence genes of nine STIs using SnapGen v3.2.1 software (San Diego, CA, USA) and OligoAnalyzer tools (Integrated DNA Technologies, IA, USA). Each pathogen probe (IDT, IA, USA) was fluorescently labeled at the 5′-end with either 5’6-FAM (Fluorescein), 5’ HEX (Hexachloro-fluorescein), or 5’ Cyanine. 5’ ROX (Carboxy-X-rhodamine) labeled probe was also used as an IAC to prevent false-negative results. Double quenchers of the probes for the nine STIs included the ZEN or TAO quencher at the ninth nucleotide to help narrow the distance between the fluorescence emitter and fluorescence quenching tip, reducing the background signal for real-time multiplex PCR, and an Iowa Black FQ/RQ as the quencher at the 3′-end. With nine pathogens of STIs, our assay consisted of three master mixes for each test. Specifically, STI master mix 1 detects GV (FAM), NG (HEX), CT (Cy5), and IAC (ROX); STI master mix 2 detects HSV-1 (FAM), CA (HEX), TV (Cy5), and IAC (ROX); STI master mix 3 detects MH (FAM), HSV-2 (HEX), MG (Cy5), and IAC (ROX) (Table 1). Positive control (PC) sequences for the nine STIs and IAC were designed (S1 Table), synthesized, and cloned into plasmid pUC19 by Phu Sa Biochem (Vietnam).

**Table 1:** Nucleotide sequences of primers and probes for nine STIs and internal control.

Pathogen name	Primer/Probe name	Sequence (5’-3’)	Length (bp)	T_m_ (°C)	Size (bp)	Gene name	Reference
***Master mix 1***							
*G*. *vaginalis*	Fw-GV	GCCATTCTTGATGCCAATCG	20	60.6	98	*tuf*	This study
	Rv-GV	GGGTGTTGATTGGGAGCTTA	20	60.7			
	P-GV	/56FAM/TGTGTTCAC/ZEN/CATCTCCGGTCGTGGT/3IABkFQ/	25	69.8			
*N*. *gonorrhoeae*	Fw-NG	CAGCATTCAATTTGTTCCGAGTC	23	61.3	91	*porA pseudogene*	Modified from [11]
	Rv-NG	CGGAACTGGTTTCATCTGATTAC	23	60.2			
	P-NG	/5HEX/ATACGCCTG/ZEN/CTACTTTCACGCTGG/3IABkFQ/	24	67.2			
*C*. *trachomatis*	Fw-CT	TTCAGTTGGGCCAGATCATG	20	61.0	73	*trpB*	Modified from [14]
	Rv-CT	CTCTTCATCGGTGGCTAATGT	21	60.8			
	P-CT	/5Cy5/AAAGGCTCG/TAO/TCCTGACTCATGCAT//3IAbRQSp/	24	66.9			
***Master mix 2***							
HSV-1	Fw-HSV1/2	GCAGTTTACGTACAACCACATACAGC	26	64	117	*UL27*	[9]
	Rv-HSV1	AGCTTGCGGGCCTCGTT	17	63.4			
	P-HSV1	/56FAM/CGGCCCAAC/ZEN/ATATCGTTGACATGGC/3IABkFQ/	25	68.2			
*C*. *albicans*	Fw-CA	GTAGAAGGTCTGCTTCGTATGG	22	60.8	100	*RPR1*	This study
	Rv-CA	GTGACTTCAAGTTCGCATATTG	22	58.8			
	P-CA	/5HEX/CCGTGGATG/ZEN/GTTGGCTGTGAGTAA/3IABkFQ/	24	67.1			
*T*. *vaginalis*	Fw-TV	AACATTGACCACACGGACAA	20	61.2	90	*Repeated DNA target*	Modified from [8]
	Rv-TV	CTTGGAACGTAAAGGCTTCTTC	22	60.2			
	P-TV	/5Cy5/TCATTTCGG/TAO/ATGGTC AGC AGCCA/3IAbRQSp/	23	67.1			
***Master mix 3***							
*M*. *hominis*	Fw-MH	TTTGGTCAAGTCCTGCAACGA	21	63.3	101	*rrnB*	[13]
	Rv-MH	CCCCACCTTCCTCCCAGTTA	20	63.8			
	P-MH	/56FAM/TACTAACAT/ZEN/TAAGTTGAGGACTCTA/3IABkFQ/	25	64.5			
HSV-2	Fw-HSV1/2	GCAGTTTACGTATAACCACATACAGC	26	64.0	117	*UL27*	[9]
	Rv-HSV2	AGCTTGCGGGCCTCGTT	17	63.4			
	P-HSV2	/5HEX/CGCCCCAGC/ZEN/ATGTCGTTCACGT/3IABkFQ/	22	69.8			
*M*. *genitalium*	Fw-MG	TTATGCGCACCAGTTACTTG	20	59.4	131	*Hypothetical protein*	Modified from [12]
	Rv-MG	AAGTTCAACTGCAGTAGTTGT	21	59.0			
	P-MG	/5Cy5/GGTGTGGAT/TAO/CGAGCGGC/3IAbRQSp/	17	63.4			
***Master mixes 1*, *2*, *3***								
Internal Amplification Control (IAC)	Fw-IAC	TGAGCGCGGCTACAGCTT	18	64.0	92	*beta-actin*	This study
	Rv-IAC	TCCTTAATGTCACGCACGATTT	22	61.6			
	P-IAC	/5ROX/CCACCACGGCCGAGCGG//3IAbRQSp/	17	68.6			

Each real-time PCR for the nine STIs contained 5 μL sample DNA, 12.5 μL 2× master mix TOPreal qPCR 2X PreMIX (Enzynomics, Incheon, Korea), 200 nM forward and reverse primers and 10–60 nM probe for pathogens, 120 nM forward and reverse primers and 120 nM probe for IAC, approximately 1,000 copies of the IAC plasmid for monitoring possible inhibition of the reactions, and nuclease-free water up to 25 μL. Real-time PCR was performed on LightCycler 96 instrument (Roche Diagnostics, Mannheim, Germany) under the following conditions: 95°C for 10 min, amplification for 45 cycles at 95°C for 10 s, 60°C for 30 s.

### Optimization of the real-time PCR assay and establishing Limit of Detection (LOD)

To optimize the real-time PCR assay conditions, serial 10-fold dilutions (10^2^ to 10^8^ copies/μL) of the control standards, i.e., the target genes of each pathogen cloned in pUC19, were used as templates. The LOD for each pathogen in the assay was determined using recombinant pUC19 plasmids carrying the target genes of the nine STIs at extremely low concentrations of 50, 10, 5, and 1 copies/reaction. For each concentration, real-time PCRs was repeated 20 times. The LOD was defined as the lowest concentration that could be detected in 95% of replicates.

### Analytical tests

#### Cross-reactivity testing

The cross-reactivity of the real-time PCR assay was determined by evaluating the amplification of specific signals for the nine targeted STIs and the production of nonspecific responses to other homologous genes from other non-targeted pathogen HPV genotypes and other common resident and contaminating bacteria in the female vagina. All assays were performed in triplicate for the reference strains/DNA controls for the nine targeted STIs (18 strains or DNA controls), non-targeted STIs (14 HPV genotypes), and non-STI bacteria (seven strains), as shown in Table 2.

**Table 2:** Analytic cross-reactivity of “in-house” real-time PCR assay for nine STIs.

STT	Micro-organism	Name/origin of strain or its DNA control	Master mix 1 (GV/NG/CT)	Master mix 2 (HSV-1/CA/TV)	Master mix 3 (MH/HSV-2/MG)
***Targeted 9 STIs***					
1	*G*. *vaginalis*	ATCC^a^ 14018	**Positive**	Negative	Negative
		PS^b^ GV DNA Control	**Positive**	Negative	Negative
2	*N*. *gonorrhoeae*	ATCC^a^ 19424	**Positive**	Negative	Negative
		BM^c^ NG DNA Control	**Positive**	Negative	Negative
3	*C*. *trachomatis*	Amplirun^d^ *Chlamydia trachomatis* DNA Control	**Positive**	Negative	Negative
		BM^c^ CT DNA Control	**Positive**	Negative	Negative
4	HSV-1	Amplirun^d^ Herpes Simplex 1 DNA Control	Negative	**Positive**	Negative
		BM^c^ HSV-1 DNA Control	Negative	**Positive**	Negative
5	*C*. *albicans*	ATCC^a^14053	Negative	**Positive**	Negative
		BM^c^ CA DNA Control	Negative	**Positive**	Negative
6	*T*. *vaginalis*	Amplirun^d^ *Trichomonas vaginalis* DNA Control	Negative	**Positive**	Negative
		PS^b^ TV DNA Control	Negative	**Positive**	Negative
7	*M*. *hominis*	Amplirun^d^ *Mycoplasma hominis* DNA Control	Negative	Negative	**Positive**
		PS^b^ MH DNA Control	Negative	Negative	**Positive**
8	HSV-2	Amplirun^d^ Herpes Simplex 2 DNA Control	Negative	Negative	**Positive**
		BM^c^ HSV-2 DNA Control	Negative	Negative	**Positive**
9	*M*. *genitalium*	Amplirun^d^ Mycoplasma Genitalium DNA Control	Negative	Negative	**Positive**
		BM^c^ MG DNA Control	Negative	Negative	**Positive**
***Non-targeted STI*: *HPV genotypes***					
1	HPV genotype 16	BM^c^ HPV16 DNA Control	Negative	Negative	Negative
2	HPV genotype 18	BM^c^ HPV18 DNA Control	Negative	Negative	Negative
3	HPV genotype 31	BM^c^ HPV31 DNA Control	Negative	Negative	Negative
4	HPV genotype 33	BM^c^ HPV33 DNA Control	Negative	Negative	Negative
5	HPV genotype 35	BM^c^ HPV35 DNA Control	Negative	Negative	Negative
6	HPV genotype 39	BM^c^ HPV39 DNA Control	Negative	Negative	Negative
7	HPV genotype 45	BM^c^ HPV45 DNA Control	Negative	Negative	Negative
8	HPV genotype 51	BM^c^ HPV51 DNA Control	Negative	Negative	Negative
9	HPV genotype 52	BM^c^ HPV52 DNA Control	Negative	Negative	Negative
10	HPV genotype 56	BM^c^ HPV56 DNA Control	Negative	Negative	Negative
11	HPV genotype 58	BM^c^ HPV58 DNA Control	Negative	Negative	Negative
12	HPV genotype 59	BM^c^ HPV59 DNA Control	Negative	Negative	Negative
13	HPV genotype 66	BM^c^ HPV66 DNA Control	Negative	Negative	Negative
14	HPV genotype 68	BM^c^ HPV68 DNA Control	Negative	Negative	Negative
***Non-STIs bacteria***					
1	*B*. *subtilis*	VTCC^e^ 11010	Negative	Negative	Negative
2	*B*. *clausii*	VTCC^e^ 11111	Negative	Negative	Negative
3	*L*. *acidophilus*	VTCC^e^ 12284	Negative	Negative	Negative
4	*L*. *casei*	VTCC^e^ 10411	Negative	Negative	Negative
5	*L*. *plantarum*	VTCC^e^ 10936	Negative	Negative	Negative
6	*L*. *rhamnosus*	VTCC^e^ 11354	Negative	Negative	Negative
7	*E*. *coli*	VTCC^e^ 12272	Negative	Negative	Negative

Notes:

^a^ATCC: standard microbial strains from American Type Culture Collection (USA);

^b^PS: DNA controls from the collection at Department of Microbiology, Hanoi Obstetrics & Gynecology Hospital (Vietnam);

^c^BM: DNA controls from the collection at Department of Microbiology, Bach Mai Hospital (Vietnam);

^d^Amplirun: DNA controls from the collection of Vircell Microbiologists (Spain);

^e^VTCC: standard microbial from Vietnam Type Culture Collection (Vietnam).

#### Assessments of agreement with commercial kits, specificity, and sensitivity

In agreement with commercial kits, the specificity and sensitivity of the “in-house” real-time PCR assay were assessed based on 136 known positive or negative reference samples of the nine STIs with a panel of positive and negative samples used in testing for each STI as described in Table 3. Commercial kits used for comparison included the following: (i) FTD STD9 kit (Fast-track Diagnostics, Junglinster, Luxembourg), (ii) Allplex CT/NG/MG/TV Assay kit (Seegene, Seoul, Republic of Korea), (iii) Allplex Vaginitis Screening Assay kit (Seegene, Seoul, Republic of Korea), and (iv) Allplex STI Essential Assay (Seegene, Seoul, Republic of Korea). Specificity (true negative rate) is the probability of a negative test being truly negative, and sensitivity (true positive rate) is the probability of a positive test being truly positive. Agreement between the real-time PCR assay and the commercial kits was assessed using Cohen’s kappa coefficient.

**Table 3:** Performance of “in-house” multiplex real-time PCR assay for STI identification compared to real-time PCR commercial kits used in hospitals.

Pathogen name	Detection by commercial real-time PCR kits		Detection by “in-house” real-time PCR assay		Sensitivity %	Specificity %	Agreement %	Cohen’s kappa coefficient
	Positive (*n*)	Negative (*n*)	Positive (*n*)	Negative (*n*)				
*G*. *vaginalis*	87	5	87	5	100	100	100	1.00
*N*. *gonorrhoeae*	12	88	12	88	100	100	100	1.00
*C*. *trachomatis*	37	60	36	61	97.4	100.0	99.0	0.98
HSV-1	16	76	16	76	100	100	100	1.00
*C*. *albicans*	36	56	36	56	100	100	100	1.00
*T*. *vaginalis*	21	86	21	86	100	100	100	1.00
*M*. *hominis*	15	90	15	90	100	100	100	1.00
HSV-2	12	80	12	80	100	100	100	1.00
*M*. *genitalium*	13	87	12	88	92.9	100	99.0	0.95
**Total**	**249**	**628**	**247**	**630**	**99.2**	**100**	**99.8**	**0.99**

#### Assessment of repeatability and reproducibility

The repeatability and reproducibility of the real-time PCR assay were evaluated by determining the coefficient of variation (CV), which was calculated using the following formula: CV = σ/μ × 100, where CV is the ratio of the standard deviation (σ) to the mean (μ) of the cycle threshold (C_t_) value for the amplification of a specific signal for each of the nine targeted STIs using recombinant DNA plasmid at 50 copies/reaction. Repeatability was assessed through intra-assay C_t_ value variation for sample of 6 replicates in one performance (*n* = 6). Reproducibility was assessed through inter-assay C_t_ value variation in 18 replicates of 3 performances (*n* = 6 × 3 = 18).

### Statistical analysis

Statistical analyses were performed using SPSS software version 20 (USA). Specificity and sensitivity were analyzed using MedCalc version 19.2.0 (MedCalc Software Ltd., Ostend, Belgium). Cohen’s kappa coefficient was determined using SPSS software (version 20, USA). LOD was analyzed using PODLOD calculation program version 9 with 95% confidence intervals (95% CI). The CV was determined using Microsoft Excel (version 16.67).

## Results

### Development of multiplex real-time PCR assay for STI detection

As shown in Table 1, our multiplex real-time PCR assay using a panel of double-quenched TaqMan probes was predesigned and developed for the simultaneous detection of nine prevalent STIs in Vietnam as follows: master mix 1 (GV/NG/CT) for *G*. *vaginalis* (GV), *N*. *gonorrhoeae* (NG), and *C*. *trachomatis* (CT); master mix 2 (HSV-1/CA/TV) for human alphaherpesvirus type 1 (HSV-1), *C*. *albicans* (CA), and *T*. *vaginalis* (TV); master mix 3 (MH/HSV-2/MG) for *Mycoplasma hominis* (MH), human alphaherpesvirus type 2 (HSV-2), and *M*. *genitalium* (MG). As shown in Table 1, all primer and probe sequences were carefully modified from references or designed in this study to meet the requirement of melting temperature (T_m_) for primers falling in the range of 60 ± 4°C and the difference in T_m_ of Fw and Rv in each primer set was less than 2°C, as well as T_m_ of >64°C for all fluorescent-labeled probes. In addition, a set of primers and a probe specifically for internal amplification control (IAC) were included in each master mix to control real-time PCR.

**Fig 1:**
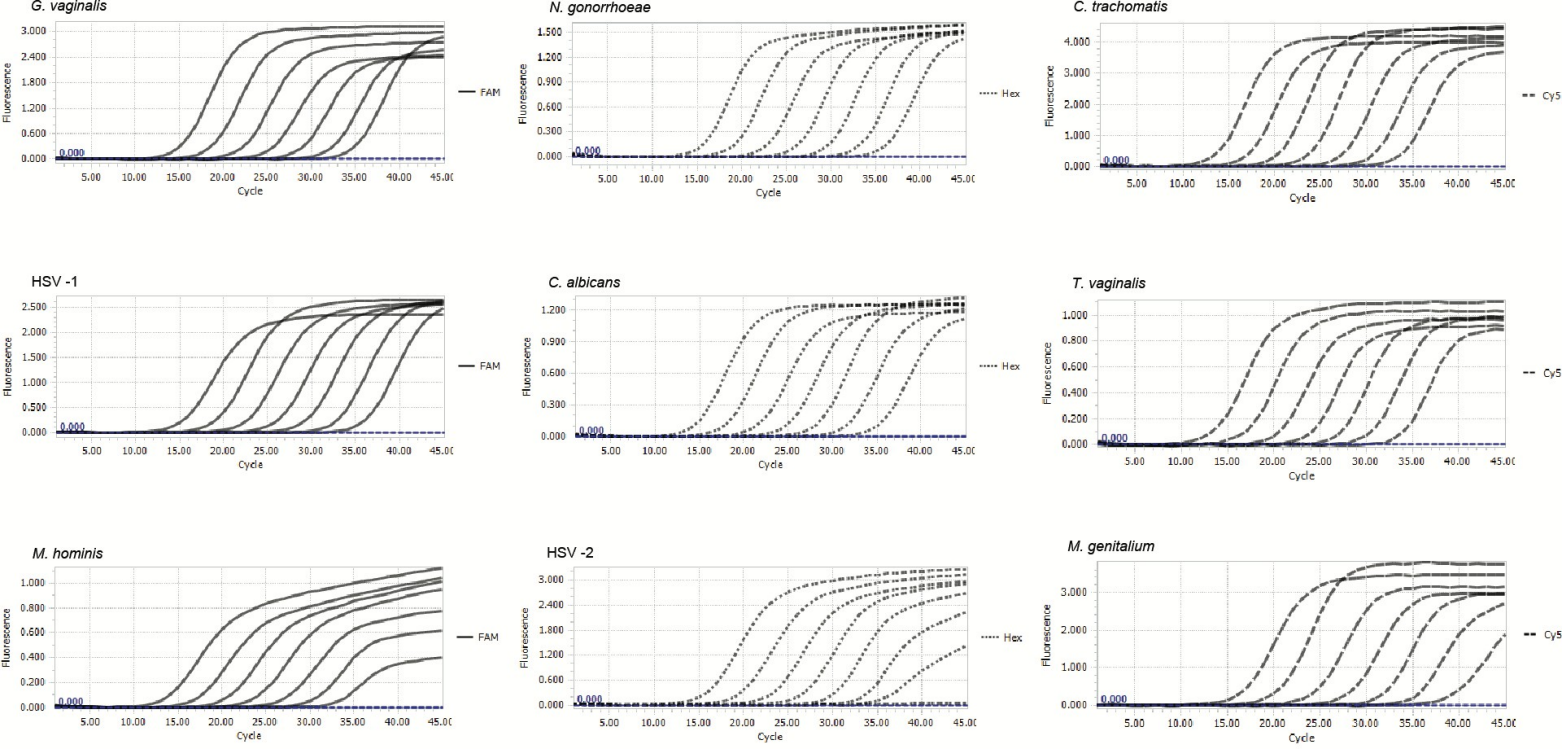
Amplification plots of “in-house” real-time PCR assay using double-quenched TaqMan probes for nine pathogens. Standard plasmids at seven 10-fold reducing concentrations in the range of 5 × 10^2^–5 × 10^8^ copies/reaction (plots were presented from the left to the right).

**Fig 2:**
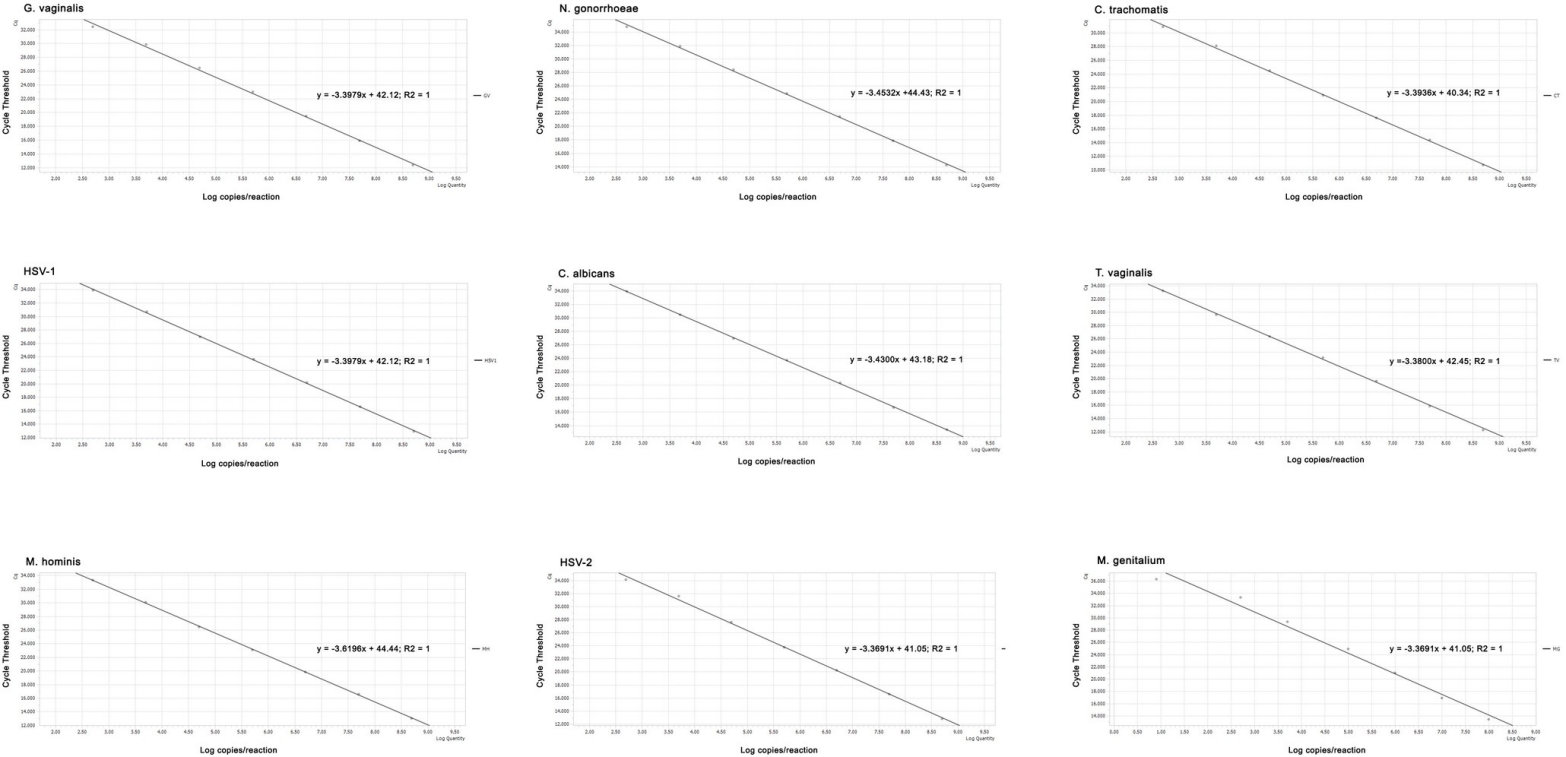
Standard curves representing for the relationship between log10 plasmid copies/reaction and the threshold cycle (C_t_). Equations: y = -ax + b with R^2^ for amplification of nine pathogens.

The three master mixes were then optimized to amplify the fluorescent signals of 5’6-FAM (Fluorescein) (for GV, HSV-1, MH), 5’ HEX (Hexachloro-fluorescein) (for NG, CA, HSV-2), 5’ Cy5 (for CT, TV, MG), and 5’ ROX (Carboxy-X-rhodamine) (for IAC) dyes. End-point fluorescence (EPF) values in the Light Cycler 96 system for the nine different target genes of nine STIs and for IAC were optimized to be >0.3 and >0.2, respectively. For data interpretation, EPF <0.2 was set up as negative signals. After extensive optimization of primer and probe sequences and their concentrations in the master mixes, we devised the master mix contents as described in Table 1 and the real-time PCR protocol as described in Materials and Methods section. In the following experiment, a serial dilution of nine cloning plasmids carrying specific target genes of nine STIs ranging from 5 × 10^8^ to 5 × 10^2^ copies/reaction was prepared to determine amplification efficiencies (E%) and correlation coefficients (R^2^) of the multiplex real-time PCR assay for each pathogen. As shown in Figs 1 and 2, there were strong linear correlations between the C_t_ values and log10 plasmid copies per reaction for all nine targeted STIs, with R^2^ and E% values in the range of 0.98–1.00 and 89%–98%, respectively.

### Analytical cross-reactivity

As listed in Table 2 and described in Materials and Methods section, a total of 18 standard microbial strains, or DNA controls of the nine STIs were used to analyze the cross-reactivity of the developed real-time PCR assay for the nine targeted pathogens. The results of the real-time PCR assay were positive for the six standard and reference strains of GV (FAM), NG (HEX), and CT (Cy5) using master mix 1 only; HSV-1 (FAM), CA (HEX), TV (Cy5) using master mix 2 only; and MH (FAM), HSV-2 (HEX), and MG (Cy5) using master mix 3 only. Positive signals had threshold cycles (C_t_) in the range of 15.89–33.83. Using the three master mixes, the amplification signals were found to be negative for all screened non-targeted 14 HPV genotypes and standard strains of 7 non-STI bacterial species that may be resident or contaminants in the female vagina. Taken together, we can conclude that there was no observable cross-reactivity among the three master mixes, and no cross-reactivity with the other 21 tested non-targeted microorganisms was found in the female vagina. The data also proved that the “in-house” real-time PCR assay was highly specific for the nine targeted STIs.

### Analytical agreement, sensitivity, and specificity

We further analyzed the agreement, sensitivity, and specificity of the developed “in-house” real-time PCR assay in comparison with the commercial kits that had CE-IVD certificates for hospital usage. The comparison experiment was conducted on 136 specimens for the “in-house” assay and (i) FTD STD9 kit (Fast-track Diagnostics, Junglinster, Luxembourg), (ii) Allplex CT/NG/MG/TV Assay kit (Seegene, Seoul, Republic of Korea), and (iii) Allplex Vaginitis Screening Assay kit (Seegene, Seoul, Republic of Korea), and (iv) Allplex STI Essential Assay (Seegene, Seoul, Republic of Korea). As shown in Table 3, the “in-house” real-time PCR assay successfully detected 87/87 GV-positive, 12/12 NG-positive, 16/16 HSV-1-positive, 36/36 CA-positive, 21/21 TV-positive, 15/15 MH-positive, and 12/12 HSV-2-positive samples. These data indicate a perfect agreement of 100% (Cohen’s kappa = 1) between the “in-house” assay and reference kits, with both sensitivity and specificity of 100.0% for detection of the seven STIs GV/NG/HSV-1/CA/TV/MH/HSV-2. In case of the two remaining STI targets, the data of 36/37 CT-positive and 12/13 MG-positive samples showed almost perfect agreement of (99.0%; Cohen’s kappa = 0.95–0.98) with the reference kits, with sensitivity and specificity of 97.4% and 100% for CT and 92.9% and 100% for MG, respectively (Table 3).

### Analytical limit of detection

To establish the limit of detection (LOD) for each pathogen, we used serial dilutions of plasmid DNA ranging from 50, 10, 5, to 1 copy/reaction of each pathogen as templates for real-time PCR. The reactions were repeated three times at a concentration of 500 copies/reaction, and 20 times at lower concentrations. As shown in Table 4, GV was positive in 19/20 (95%) cases, while the three STIs, including NG, CA, and TV, were positive in 20/20 (100%) cases at a concentration of 10 copies/reaction. In contrast, the five STIs, CT, HSV-1, MH, HSV-2, and MG, were positive in 20/20 (100%) cases at a higher concentration of 50 copies/reaction. At a lower concentration of 10 copies/reaction, only 0%–85% of cases were detected for these five pathogens, depending on each target gene. Using PODLOD calculation program version 9, the LOD was estimated to be 17.1 copies/reaction (95% CI: 11.69–25.15) for GV, 18.6 copies/reaction (95% CI: 12.62–27.32) for NG, 37.2 copies/reaction (95% CI: 24.86–55.81) for CT, 22.8 copies/reaction (95% CI: 15.36–22.84) for HSV-1, 15.5 copies/reaction (95% CI: 10.58–22.60) for CA, 8.2 copies/reaction (95% CI: 5.56–12.19) for TV, 24.5 copies/reaction (95% CI: 16.49–36.53) for MH, 48.3 copies/reaction (95% CI: 32.27–72.40) for HSV-2 and 57.8 copies/reaction (95% CI: 38.56–86.75) for MG. In conclusion, the LOD was estimated to be within the range of 8–58 copies/reaction for the nine STIs.

**Table 4:** Limit of detection of “in-house” multiplex real-time PCR assay.

Pathogen name	No. of replicates (%) for positive standards of real-time PCR					LOD, copies/reactions (95% CI)
	500 copies	50 copies	10 copies	5 copies	1 copy	
*G*. *vaginalis*	3/3 (100%)	20/20 (100%)	**19/20 (95%)**	9/20 (45%)	2/20 (10%)	17.1 (11.69–25.15)
*N*. *gonorrhoeae*	3/3 (100%)	20/20 (100%)	**20/20 (100%)**	7/20 (35%)	1/20 (5%)	18.6 (12.62–27.32)
*C*. *trachomatis*	3/3 (100%)	**20/20 (100%)**	10/20 (50%)	8/20 (40%)	0/20 (0%)	37.2 (24.86–55.81)
HSV-1	3/3 (100%)	**20/20 (100%)**	13/20 (65%)	12/20 (60%)	2/20 (10%)	22.8 (15.36–33.84)
*C*. *albicans*	3/3 (100%)	20/20 (100%)	**20/20 (100%)**	10/20 (50%)	1/20 (5%)	15.5 (10.58–22.60)
*T*. *vaginalis*	3/3 (100%)	20/20 (100%)	**20/20 (100%)**	17/20 (85%)	4/20 (20%)	8.2 (5.56–12.19)
*M*. *hominis*	3/3 (100%)	**20/20 (100%)**	17/20 (85%)	7/20 (15%)	0/20 (0%)	24.5 (16.49–36.53)
HSV-2	3/3 (100%)	**20/20 (100%)**	10/20 (50%)	3/20 (15%)	0/20 (0%)	48.3 (32.27–72.40)
*M*. *genitalium*	3/3 (100%)	**20/20 (100%)**	7/20 (35%)	3/20 (15%)	0/20 (0%)	57.8 (38.56–86.75)

### Analytical repeatability and reproducibility

To validate the precision and accuracy of the real-time assay, we analyzed the repeatability and reproducibility for each of the nine targeted STIs at a concentration of 50 copies/reaction, as described in the Materials and methods. All replicates yielded amplification signals for the nine pathogens, with mean C_t_ and SD values of 34–37 and 0.2–1.0, respectively. The repeatability and reproducibility coefficients of variation (CV) for the nine pathogens were less than 3%, indicating excellent repeatability and reproducibility (Table 5).

**Table 5:** Repeatability and reproducibility of the “in-house” multiplex real-time PCR assay.

Pathogen	Repeatability of C_t_ value									Reproducibility of C_t_ value		
	Batch 1			Batch 2			Batch 3					
	Mean 1	SD 1	CV 1 (%)	Mean 2	SD 2	CV 2 (%)	Mean 3	SD 3	CV 3 (%)	Mean	SD	CV (%)
*G*. *vaginalis*	36.17	0.91	2.52	36.56	0.86	2.35	36.53	0.98	2.68	36.42	0.21	0.58
*N*. *gonorrhoeae*	37.37	0.74	1.99	37.10	0.86	2.32	37.83	1.02	2.71	37.43	0.37	0.99
*C*. *trachomatis*	35.38	0.65	1.84	35.83	0.84	2.34	35.09	0.55	1.57	35.44	0.37	1.05
HSV-1	36.94	0.58	1.58	36.84	0.58	1.58	37.33	1.01	2.71	37.04	0.26	0.70
*C*. *albicans*	37.20	0.53	1.41	37.44	0.34	0.92	37.06	1.12	3.01	37.23	0.19	0.51
*T*. *vaginalis*	36.09	0.89	2.46	36.20	0.48	1.31	35.98	0.67	1.85	36.09	0.11	0.30
*M*. *hominis*	36.43	0.34	0.92	35.88	0.29	0.80	36.05	0.21	0.60	36.12	0.28	0.78
HSV-2	35.41	0.77	2.16	35.26	0.64	1.83	35.28	0.72	2.05	35.32	0.08	0.22
*M*. *genitalium*	35.59	0.94	2.64	34.80	0.54	1.56	34.78	0.46	1.33	35.05	0.46	1.32

CV, coefficient of variation; SD, standard deviation.

### Turnaround time and costs

The total turnaround time for this assay was approximately 2 h. The assay began with a 30 min DNA extraction, followed by approximately 1.5 h for real-time PCR amplification and data analysis. The cost of the three master mixes for the “in-house” real-time PCR assay was only 2.34 USD (S2 Table), which was approximately 8-fold cheaper than that of the commercial kits. These data and cost analyses clearly demonstrate the time-saving and cost-effectiveness of our real-time PCR double-quenched TaqMan probe assay for the detection of common STIs in Vietnamese women. In the following experiments, we applied our “in-house” assay to the rapid screening of clinical specimens obtained from female patients from hospitals and a CDC located in Northern Vietnam.

### Profile of STIs prevalence in women who visited hospitals in Northern Vietnam

**Fig 3:**
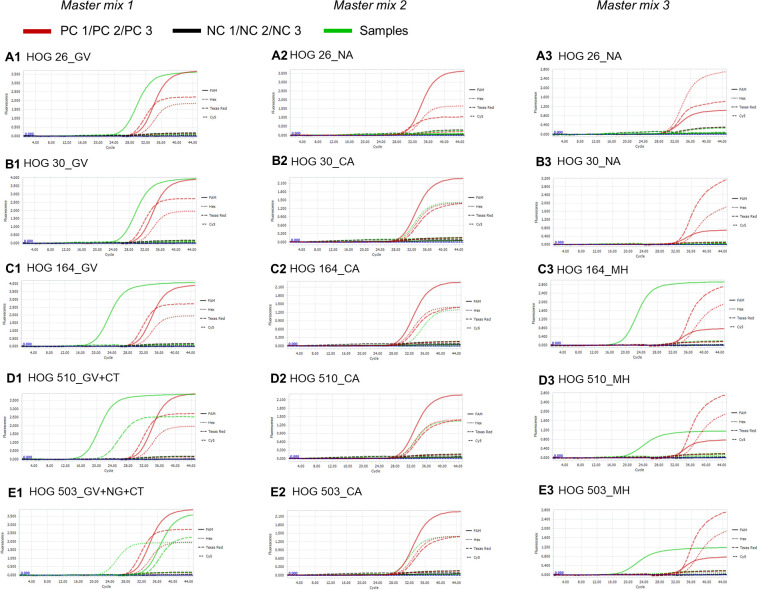
Real-time PCR amplification curves taken from five representative vaginal swab clinical samples. Samples include positive with one (A1–A3), two (B1–B3), three (C1–C3), four (D1–D3), and five (E1–E3) pathogens among the screened nine STIs. Positive and negative controls in the three master mixes 1/2/3 were PC 1/2/3 and NC1/2/3, respectively. Amplification curves for positive controls, negative controls, and positive clinical samples are presented in red, black, and green, respectively.

We recruited 535 women aged 18–66 years who were living in Northern Vietnam and who underwent gynecological examination at Bach Mai Hospital and Hanoi Obstetrics and Gynecology Hospital; 510/535 cases (95.33%) were women of reproductive age (18–49 years old); 502/535 (93.83%) had typical clinical symptoms of vaginal infections (itching, heavy vaginal odor, vaginal bleeding, and vaginal discharge), with a high degree of inflammation, indicated by high leukocyte densities. To investigate the prevalence of STIs in these patients, we screened all vaginal swab samples by using an “in-house” multiplex real-time PCR double-quenched TaqMan probe assay. Representative real-time PCR TaqMan probe amplification curves for vaginal swab samples that were positive for one (GV, Fig 3A1–3A3), two (GV/CA, Fig 3B1–3B3), three (GV/CA/MH, Fig 3C1–3C3), four (GV/CT/CA/MH, Fig 3D1–3D3), and five (GV/NG/CT/CA/MH, Fig 3E1–3E3) pathogens are shown in Fig 3.

Notably, up to 532 cases (99.44%) were positive for at least one of the nine STIs, whereas only 3/535 (0.56%) of the cases were undetectable (Table 6). Regarding individual or multiple infections, we found that there were 202/535 (37.76%) samples infected with a single pathogen, including 181/535 (33.83%) GV, 19/535 (3.55%) CA, and 1/535 (0.19%) infected with either NG or MH. We also detected 248/535 (46.36%) patients infected with two pathogens simultaneously, in which the combination of GV and CA with 204/535 (38.13%) was the highest, followed by the combination of GV + CT and/or GV + MH with 3.74% and 2.06%, respectively. As shown in Table 6, the other six combinations of GV and CA with other pathogens were less common and accounted for only 0.19%–2.43%. Patients infected with three, four, and five pathogens were also detectable and accounted for 82/535 cases (15.32%). Of these, 63/535 (11.78%) patients were positive for three STIs with common combinations of GV, CA, and MH and/or GV, CT, and CA with 3.93% and 3.55%, respectively. Furthermore, 16/535 (2.99%) patients were positive for four STIs, including 6/535 (1.12%) cases positive for GV, CT, CA, and MH; 4/535 (0.75%) cases positive for GV, NG, CT, and CA; and six other less common combinations with the presence of TV, MG, HSV-1, and HSV-2. Patients who were positive for multiple pathogens accounted for 61.68% (330/535 cases), which was 1.63-fold higher than the number of patients infected with a single pathogen. Three cases were positive for five pathogens simultaneously, including two cases (0.37%) carrying combinations of GV, NG, CT, CA, and MH; and one case (0.19%) carrying a combination of GV, NG, CT, MG, and HSV-2. Taken together, the data on STIs obtained from vaginal swab samples in Vietnamese women indicate that our novel real-time PCR double-quenched TaqMan probe assay is reliable for the detection of most of the major STIs circulating in Vietnam, with a high prevalence of single and multiple pathogens.

**Table 6:** Prevalence of STIs that were screened using multiplex real-time PCR TaqMan probe.

Characteristic of STIs	Number of cases	Rate (%)
***Negative with STIs***	***3***	***0*.*56***
***Positive with one STI***	***202***	***37*.*76***
*G*. *vaginalis*	181	33.83
*C*. *albicans*	19	3.55
*N*. *gonorrhoeae*	1	0.19
*M*. *hominis*	1	0.19
***Positive with two STIs***	***248***	***46*.*36***
*G*. *vaginalis* and *C*. *albicans*	204	38.13
*G*. *vaginalis* and *N*. *gonorrhoeae*	1	0.19
*G*. *vaginalis* and *C*. *trachomatis*	20	3.74
*G*. *vaginalis* and *T*. *vaginalis*	2	0.37
*G*. *vaginalis* and *HSV-1*	2	0.37
*G*. *vaginalis* and *M*. *hominis*	11	2.06
*C*. *albicans* and *N*. *gonorrhoeae*	2	0.37
*C*. *albicans* and *M*. *hominis*	4	0.75
*C*. *albicans* and *M*. *genitalium*	2	0.37
***Positive with three STIs***	63	11.78
*G*. *vaginalis*, *N*. *gonorrhoeae and C*. *trachomatis*	6	1.12
*G*. *vaginalis*, *C*. *trachomatis* and *C*. *albicans*	19	3.55
*G*. *vaginalis*, *C*. *trachomatis* and *HSV-1*	1	0.19
*G*. *vaginalis*, *C*. *trachomatis* and *HSV-2*	1	0.19
*G*. *vaginalis*, *N*. *gonorrhoeae and C*. *albicans*	5	0.93
*G*. *vaginalis*, *C*. *trachomatis* and *M*. *hominis*	4	0.75
*G*. *vaginalis*, *C*. *albicans* and *HSV-1*	2	0.37
*G*. *vaginalis*, *C*. *albicans* and *T*. *vaginalis*	2	0.37
*G*. *vaginalis*, *C*. *albicans* and *M*. *hominis*	21	3.93
*G*. *vaginalis*, *C*. *albicans* and *M*. *genitalium*	1	0.19
*G*. *vaginalis*, *T*. *vaginalis* and *M*. *hominis*	1	0.19
***Positive with four STIs***	16	2.99
*G*. *vaginalis*, *N*. *gonorrhoeae*, *C*. *trachomatis and C*. *albicans*	4	0.75
*G*. *vaginalis*, *N*. *gonorrhoeae*, *C*. *trachomatis and HSV-1*	1	0.19
*G*. *vaginalis*, *N*. *gonorrhoeae*, *C*. *trachomatis* and *M*. *hominis*	1	0.19
*G*. *vaginalis*, *N*. *gonorrhoeae*, *C*. *albicans and M*. *hominis*	1	0.19
*G*. *vaginalis*, *C*. *trachomatis*, *C*. *albicans and M*. *hominis*	6	1.12
*G*. *vaginalis*, *C*. *albicans*, *M*. *hominis and HSV-1*	1	0.19
*G*. *vaginalis*, *T*. *vaginalis*, *M*. *hominis and HSV-2*	1	0.19
*G*. *vaginalis*, *T*. *vaginalis*, *M*. *hominis and M*. *genitalium*	1	0.19
***Positive with five STIs***	3	0.56
*G*. *vaginalis*, *N*. *gonorrhoeae*, *C*. *trachomatis*, *C*. *albicans* and *M*. *hominis*	2	0.37
*G*. *vaginalis*, *N*. *gonorrhoeae*, *C*. *trachomatis*, *M*. *genitalium* and *HSV-2*	1	0.19
**Total**	**535**	**100**

## Discussion

Successful treatment of STDs requires rapid, sensitive screening for multiple STIs in vaginal swab samples to select the appropriate pharmacological therapy; thus, lowering the risk of developing antibiotic resistance and pathogen transmission and reducing the public health impact. However, the most recognized routine tests used to identify STIs in developing or middle-income countries have either limited sensitivity (microscopic inspection) or lengthy turnaround time (microbial culture). Molecular tests based on DNA-hybridization mini arrays or real-time PCR for screening various STIs on a large scale are applicable in terms of reliability and short turnaround time, but the prices of commercially available kits are unaffordable, especially in limited resource areas. To address this limitation, several research groups have attempted to devise improved real-time PCR assays with high specificity and sensitivity for “in-house” usage. A number of studies have developed molecular tests for single-pathogen detection [8, 9, 11–13, 25], while other studies have focused on the development of multiplex real-time PCR to detect two or three pathogens simultaneously in a single assay [14, 26–28]. One of the more notable attempts is a multiplex real-time PCR melting curve assay method for the simultaneous detection of up to nine STI pathogens, including CT, NG, MG, TV, MH, UU, UP, HSV-1, and HSV-2, as demonstrated by Hu et al. (2019) [29]. Such an approach would require sophisticated optimization of the probes to achieve a distinguishable melting temperature for each target with the same fluorescence signal.

To overcome this difficulty, both in probe design and practical melting curve analysis, we created a new real-time PCR double-quenched TaqMan probe assay for the detection of nine common STIs circulating in Vietnam. Because of the reduced background of the double-quenched TaqMan probe, four probes were included in a multiplex real-time PCR experiment. The four fluorescent dyes FAM, HEX, Cy5, and ROX were chosen in this predesigned panel of probes because of their popularity in order-made synthesis and their emission wavelength being separated well under four optical channels of open real-time PCR systems, such as Light Cycler 96/480 (Roche Diagnostics, Germany), CFX96 Touch Real-Time PCR (Bio-Rad, USA), and 7500-Fast Real-time PCR (Thermo Fisher Scientific, USA), which are commonly used in hospitals to ensure the applicability of the assay in a wide range of clinical settings in a middle-income country. Because the annealing temperature (T_m_) of primers and probes for each master mix was optimized for conducting all three reactions concurrently under one real-time PCR condition, the detection throughput and turnover rates were successfully increased. Our optimized panel of probes was able to efficiently bind and detect any of the nine STIs in all of the 92 evaluated samples with a high specificity of 100% and perfect agreement of 98.8%–100% with commercial imported CE-IVD certificate kits that are being used in several central hospitals. Although the number of positive reference samples for some pathogens, such as *N*. *gonorrhoeae*, *T*. *vaginalis*, *M*. *hominis*, and *M*. *genitalium*, was limited due to their low infection rates, we were able to calculate the sensitivity and specificity, which were high, with Cohen’s kappa coefficient values of 0.95–1, in good agreement with the results from commercial kits. Future studies should include a larger number of reference samples to confirm the reliability of this analytical test. The analytical sensitivities of our assay were 100% for seven pathogens (GV, NG, HSV-1, CA, TV, MH, HSV-2) and 92.9%–97.4% for MG/CT with the LODs of 8–58 copies/reaction for the nine pathogens. The high sensitivities and low LODs are probably due to the role of ZEN or TAO quenchers labeled at the ninth nucleotide from the 5′-end in the double-quenched TaqMan probes, and are critical for detecting STIs at an early stage of infection or when sample size is restricted. When comparing the quality of our “in-house” assay to commercial kits, we admit that the 58 copies/reaction LOD of MG in our assay is slightly less sensitive than the 10–50 copies/reaction LODs of Anyplex II STI-7 Detection (CE-IVD, Seegene, Seoul, Republic of Korea), as reported by the manufacturer kit. Nevertheless, our LODs for the nine STIs were greatly improved compared to the LOD of 200 copies/reaction in the real-time PCR assay for the simultaneous detection of nine pathogens reported by Hu et al. [29]. Further optimization of TaqMan probes with better quantum yield and photostable fluorescent dyes may have assisted in achieving higher sensitivities and lower LODs; however, such an increase in sensitivity and a reduction in LODs would necessarily increase the overall cost per reaction, limiting their utility in low-middle-income nations. Similarly, using superior Taq polymerase with higher 5′-exonuclease activity and higher heat stability would significantly increase the total cost per reaction. When scaled up as a routine test in a clinical setting, these cost increases would cumulatively interfere with the assay’s accessibility. Regarding the repeatability and reproducibility of real-time PCR assays for STI detection, only one study by Muller et al., 2012 [30] reported CVs for real-time PCR detection of an STI, with values of 0.19–2.01 when testing for MH using serial (10-fold) dilutions (10–10^4^ copies/reaction). Our CVs for repeatability (0.60–0.92) and reproducibility (0.78) for MH at 50 copies/reaction are comparable to those in Muller’s study (0.44–2.01 and 0.75–1.19 at 10–100 copies/reaction, respectively). Although we have not yet determined the CVs at lower concentrations, such as 5–10 copies/reaction, CVs of less than 3% were obtained for all nine STIs at 50 copies/reaction, indicating that the assay is highly repeatable and reproducible.

We also calculated that the cost of reagents and consumables for one real-time PCR test of nine STI detection was 2.34 USD (S2 Table), which was significantly cheaper than the around 12–15 USD/test spent for imported commercial kits. This cost analysis was estimated using real-world prices for reagents and consumables from the specific brands used in this study, which may not be universally applicable. Therefore, we chose to employ the given double-quenched probes and real-time PCR master mix because of our restricted research budget and clinical unit requirements.

This optimized detection assay was then applied to a clinical cohort of vaginal samples collected from patients visiting two central hospitals in Northern Vietnam for vaginal examination. Each real-time PCR performance per thermal cycler took just 2 h, including 1.5 h for thermal cycling and an additional 30 min for nucleic acid extraction, which allowed the rapid screening of 535 samples. We detected all nine STIs in these samples and observed their overall prevalence to be as high as 99.44%, with 37.76% and 46.36% samples infected with single and double pathogens, respectively. Samples infected with three, four, and five pathogens accounted for the remaining 14.8%. The prevalence of the nine STIs in decreasing order was as follows: GV, CA, CT, MH, NG, TV, HSV-1, MG, and HSV-2, as shown in Table 6. Among them, the most prevalent single pathogen was GV (33.83%) and the most prevalent dual combination was GV + CA (38.13%) found in Vietnamese women who visited hospitals for STD treatment. These high rates of GV and its co-infections were similar to a recent report of STIs with the highest prevalence of 36% GV in Lebanon women, which has been reported by Hanna et al. [31] but were 2- to 4-fold higher than those in women living in the West Region, Cameroon, with the most prevalent 25.57% CA 17.08% GV, and 9.49% CA + GV [32]. In addition to the most prevalent GV infection rates, the other three common pathogens that were found to infect Vietnamese women’s vagina causing single or multiple infections, included CA, CT, and MH, with 55.14%, 12.34%, and 10.28%, respectively. These three rates were relatively close to those of previous studies regarding the 64% CA infection rate found in Nigerian women [33], 8.8% CT infection rate found in Chilean adolescents [34], and approximately 11% of MH infection rates found in Brazilian women without gynecologic complaints and at reproductive age [35] and in gynecological outpatients of a tertiary hospital in China from 2015 to 2018 [36]. The rate of MH in our study was lower than that of 17.34% in Tanzanian women [37] but higher than that of 3.39% in infertile women in Romania’s Northeast Region [38]. Our data differed from the results of a study on the urogenital tract of Brazilian women, with the highest rates of STIs being MH (31.8%) and MG (28.1%), followed by TV, GV, NG, and CT with prevalence of 3.0%, 21.5%, 42.4%, and 1.7%, respectively [35]. In another retrospective study by Sarier 2019 [39], in which common STIs were detected in 171 Turkish patients with urethritis using a commercial real-time PCR assay (ELITechGroup, Inc.), the STI distribution differed from the distribution in our study and was as follows: CT (22.9%), NG (21.7%), GV (16.8%), MG (10.5%), MH (3.7%), TV (3.1%), CA (1.8%), and HSV-2 (1.2%), with 14% and 2.9% of patients infected with two and three pathogens, respectively. The percentages of patients infected with both NG and non-NG pathogens and NG only were much higher than ours, at 17.3% and 82.7%, respectively.

To date, there have been a number of reports of patients co-infected with 2–3 STIs pathogens [32, 34, 36, 38–42]. However, to the best of our knowledge, there have been no publications showing patients co-infected with 4–5 pathogens. For the first time, we detected eight types of STI quad-pathogenic combinations, including GV/NG/CT/CA, GV/NG/CT/HSV-1, GV/NG/CT/HM, GV/NG/CA/HM, GV/CA/MH/HSV-1, GV/TV/MH/HSV-2, and GV/TV/MH/HG, and two types of STI penta-pathogenic combinations: GV/NG/CT/CA/MH and GV/NG/CT/MG/HSV-2. Treatment in these cases is complicated due to multiple infection. For the patient infected with the five pathogenic combination GV/NG/CT/MG/HSV-2, the following treatment plan recommended by doctors at Hanoi Obstetrics and Gynecology Hospital (Hanoi, Vietnam) should be considered: (1) priority treatment for NG infection with either intramuscular or oral beta-lactam antibiotics (e.g., ceftriaxone or cefoxitin) because the symptoms caused by NG are the most severe; (2) parallel treatment for CT infection with oral tetracycline or macrolide antibiotics (e.g., doxycycline or azithromycin); (3) parallel treatment for MG and GV infections using metronidazole antibiotics as a vaginal-capsule formulation; and (4) periodic health monitoring, assessment of vaginal lesions, and follow-up to assess progression to vaginal chronic inflammation or cancer for HSV-2 infection. Oral administration of the antiviral acyclovir is recommended only in patients exhibiting obvious clinical symptoms of HSV infection. According to the findings, the emergence of multiple complex STIs in Vietnamese women is concerning, and multiplex-STI detection by real-time PCR assay may provide doctors with more appropriate therapy plans for efficient treatment of these complicated cases.

When all factors including cross-reactivity, sensitivity, specificity, LOD, agreement with CE-IVD commercial kits, repeatability, reproducibility, turnaround time, and cost are considered, we believe that our “in-house” real-time PCR assay is an effective alternative to expensive imported commercial kits. When used in conjunction, our assay allows for the simultaneous detection of common STIs with high sensitivities and low LODs, making it a potential tool for the effective treatment of STDs in developing countries, particularly in non-hospitalized patients who require their results within half a day before beginning treatment. This assay can be used not only to screen for the presence of STIs but also to measure the microbial loads of the nine STIs in clinical samples, particularly opportunistic pathogens, for which treatment decisions may be based not only on positive or negative results but also on microbial load. Furthermore, we believe that the assay design and development technique provided here might serve as a paradigm for the development of comparable detection assays for other STIs that are currently prevalent in different regions.

## Supporting information

S1 Table Nucleotide sequences of the positive controls for the nine STIs and internal amplification control (IAC). (PDF)

S2 Table Cost of one reaction for the nine STI real-time PCR assay. (PDF)

S1 File Informed consent form. (PDF)
