# Supplementary material for: Simultaneous real-time PCR detection of nine prevalent sexually transmitted infections using a predesigned double-quenched TaqMan probe panel
Source: PLoS One. 2023 Mar 6;18(3):e0282439. doi: 10.1371/journal.pone.0282439 (PMC9987813; doi:10.1371/journal.pone.0282439)
Supplement: S1 Table — (PDF) [file pone.0282439.s001.pdf]

S1 Table. Nucleotide sequences of the positive controls for the nine STIs and internal amplification control (IAC)

No.	PC name	Size (bp)	Sequence (5'-3')
1	*G. vaginalis*	98	GCCATTCTTGATGCCAATCGAAGATGTGTTCACCATCT CCGGTCGTGGTACCGTTGTCACCGGTCGTGTTGAGCGT GGTAAGCTCCCAATCAACACCC
2	*N. gonorrhoeae*	91	CAGCATTCAATTTGTTCCGAGTCAAAACAGCAAGTCCG CCTATACGCCTGCTACTTTCACGCTGGAAAGTAATCAG ATGAAACCAGTTCCG
3	*C. trachomatis*	73	TTCAGTTGGGCCAGATCATGCCGAAATGCATGAGTCAG GACGAGCCTTTTATACATTAGCCACCGATGAAGAG
4	HSV-1	117	GCAGTTTACGTACAACCACATACAGCGCCATGTCAACG ATATGTTGGGCCGCGTTGCCATCGCGTGGTGCGAGCTG CAGAATCACGAGCTGACCCTGTGGAACGAGGCCCGCA AGCT
5	*C. albicans*	100	GTAGAAGGTCTGCTTCGTATGGGAATGGCGCCGTGGAT GGTTGGCTGTGAGTAATTCTTTACTACAAGCTGTTTAG TGCAATATGCGAACTTGAAGTCAC
6	*T. vaginalis*	90	AACATTGACCACACGGACAAAAAGTGTCATTTCGGAT GGTCAAGCAGCCAATCGCATTCGAGCACTTCGAAGAA GCCTTTACGTTCCAAG

7	*M. hominis*	101	TTTGGTCAAGTCCTGCAACGAGCGCAACCCCTATCTTT AGTTACTAACATTAAGTTGAGGACTCTAGAGATACTGC CTGGGTAACTGGGAGGAAGGTGGGG
8	HSV-2	117	GCAGTTTACGTATAACCACATACAGCGCCACGTGAACG ACATGCTGGGGCGCATCGCCGTCGCGTGGTGCGAGCTG CAGAACCACGAGCTGACTCTCTGGAACGAGGCCCGCA AGCT
9	*M. genitalium*	131	TTATGCGCACCAGTTACTTGAAAAAAATACCCATAATG AATAGTGATAGTGATCTAAAACTCCAAAAGGTGTGGA TCGAGCGGCATGTTGATCAAGATGAACTTAGTTTAACA ACTACTGCAGTTGAACTT
10	IAC	92	TGAGCGCGGCTACAGCTTCACCACCACGGCCGAGCGG GAAATCGTGCGTGACATTAAGGAAAGGGCGAATTCTG CAGATATCCATCACACTG
