# Supplementary material for: Simultaneous real-time PCR detection of nine prevalent sexually transmitted infections using a predesigned double-quenched TaqMan probe panel
Source: PLoS One. 2023 Mar 6;18(3):e0282439. doi: 10.1371/journal.pone.0282439 (PMC9987813; doi:10.1371/journal.pone.0282439)
Supplement: S2 Table — (PDF) [file pone.0282439.s002.pdf]

S2 Table. Cost of one reaction for the nine STI real-time PCR assay

**Reagents and consumables of one reaction**	**Cost (US dollar)**
One master mix	***0.78***
12.5 µL Real time PCR master mix[a]	*0.44*
Primers[b]	-
PCR tube[c]	*0.10*
FAM and HEX labelled double-quenched TaqMan probes[d]	*0.08*
Cy5 and ROX labelled double-quenched TaqMan probes[e]	*0.16*
**One reaction consisting of 3 master mixes (x 3)**	**2.34**

Notes:

[a] *TOPreal™ qPCR 2XPreMIX (Enzynomics, Incheon, Korea): $176 for 5 mL.*

[b] *The cost of primers (PHUSA Biochem, Can Tho, Vietnam) is almost negligible.*

[c] *PCR tube (Thermo Scientific, MA, USA): $100 for 1000 tubes.*

[d] *FAM labelled double-quenched TaqMan probe (IDT, IA, USA): $320 for 8000 reactions; HEX labelled double-quenched TaqMan probe (IDT, IA, USA): $320 for 8000 reactions.*

[e] *Cy5 labelled double-quenched TaqMan probe (IDT, IA, USA): $640 for 8000 reactions; ROX labelled probe (IDT, IA, USA): $640 for 8000 reactions.*
