# Supplementary material for: Simultaneous real-time PCR detection of nine prevalent sexually transmitted infections using a predesigned double-quenched TaqMan probe panel
Source: PLoS One. 2023 Mar 6;18(3):e0282439. doi: 10.1371/journal.pone.0282439 (PMC9987813; doi:10.1371/journal.pone.0282439)
Supplement: S1 File — (PDF) [file pone.0282439.s003.pdf]

S1 File. Informed consent form

This informed consent form is for patients/ consent to join research:

Name of Principle Investigator: Assoc. Prof. Bui Thi Viet Ha

Name of Organization: VNU University of Science

Name of Project and Version: Development of real-time PCR based assay for detecting and screening the most common sexual transmission infections

This Informed Consent Form (ICF) has two parts:

- Information Sheet (to share information about the study with you)
- Certificate of Consent (for signatures if you choose to participate)

You will be given a copy of the full Informed Consent Form

Part I: Information Sheet

Introduction

I am Bui Thi Viet Ha and I am conducting research on "Development of real-time PCR based assay for detecting and screening the most common sexual transmission infections", under the supervision of VNU University of Science and Dinh Tien Hoang Institute of Medicine. I am going to give you information and invite you to be part of this research. You do not have to decide today whether will participate in the research. Before you decide, you can talk to anyone you feel comfortable with about the research.

This consent form may contain words that you do not understand. Please ask me to stop as we go through the information, and I will take time to explain. If you have questions later, you can ask me or another researcher.

Purpose of the research

Sexually transmitted diseases (STDs) account for the worldwide burden on public health and medical care. STDs can cause infertility, ectopic pregnancy, and genital neoplasia. According to the World Health Organization, more than 1 million sexually transmitted infections (STIs) are acquired every day worldwide, with an estimated 374 million new infections each year (https://www.who.int/news-room/fact-sheets/detail/sexually-transmitted-infections-(stis)). STDs are caused by various microorganisms such as bacteria, viruses, yeast, and protozoa. Common STIs include, but are not limited to, *Chlamydia trachomatis, Neisseria gonorrhoeae*, *Candida albicans*, *Mycoplasma genitalium*, *Trichomonas vaginalis*, *Mycoplasma hominis*, *Gardnerella vaginalis*, and herpes virus simplex types 1 and 2. In Vietnam, the prevalence of these nine STIs is relatively high.[1-4] Unfortunately, infections caused by multiple STDs often present similar clinical symptoms, making the clinical differentiation of pathogens very difficult. Therefore, it is critical to create a sensitive, multitarget, and high-throughput approach for detecting common STIs. Furthermore, rapid and accurate diagnosis is essential to prevent further transmission, provide urgent intervention, implement early treatment, and lower patient care expenses. Cell culture, microscopic inspection, enzyme immunoassay, and other serological techniques are

common STIs diagnostic algorithms; however, they have certain limitations in terms of sensitivity and a long turnaround time.

Real-time polymerase chain reaction (PCR) is more advantageous because they offer a shorter turnaround time than the conventional methods. Although several commercial multiplex real-time PCR kits have been developed so far, they are expensive and do not cover the major nine STIs found in Vietnam, which makes them unsuitable for screening purposes in hospitals and centers for disease control (CDCs) in developing countries. Therefore, this study focuses on our efforts to develop a sensitive simultaneous real-time PCR assay utilizing double-quenched TaqMan probes for the detection of nine STD pathogens commonly found in Vietnam and may also be found in other countries. This "in-house" real-time PCR assay can be regarded as a model for the development of other panels suited for screening prevalent STIs in countries where the economic and epidemiological status does not allow for large-scale usage of imported kits.

Voluntary Participation

Your participation in this research is entirely voluntary. You are free to choose whether or not to participate in this study. You should consider this carefully before deciding to participate. The choice that you make will have no bearing on the health care we provide. Please feel free to ask the doctor who is talking to you about this consent form with any questions that come to your mind. If you agree to participate in this study, you will be asked to sign or fingerprint the last page of this form.

Right to Refuse or Withdraw

You do not have to take part in this research if you do not wish to do so. The doctors will respect your decision and will still treat you according to routine hospital practice. The same goes for if you give your consent to participate in the study but then you change your mind. You are free to refuse now or later, and this should not affect the medical care your child receives.

Reimbursements

You will not be provided any incentive to take part in the research.

Procedures

- Time: The research was carried out over a 19-month period, from December 2020 to July 2022.
- Location: Bach Mai Hospital (BMH), Hanoi Obstetrics & Gynecology Hospital (HOGH).
- Number of participants: 500-600 people.
- Research steps:

Step 1: The researchers provide study information.

Step 2: If you agree, please sign the ICF (if you don't agree, you can stop here).

Step 3: You are interviewed at the hospital you are visiting.

Step 4: At the hospital, you will be examined by gynecologists and taken your vaginal swab sample for sexual transmission infections (STIs) testing.

Step 5: Your specimens will be screened for STIs at VNU University of Science by the "in-house" developed real-time PCR assay.

Step 6: The test results will be emailed to you, or you can pick them up at BMH or HOGH.

Benefits

- Besides the routine examination for obstetrics and gynecology, and sexually transmitted diseases, you are advised on methods of prevention and treatment of sexually transmitted diseases (if present).

- You will be screened for STIs for free; the results of this test will indicate whether or not you are infected with STIs or not. If this is the case, you will be advised on treatment therapy.

Risks

You may experience some discomfort during the physical exam, but it should disappear quickly.

Confidentiality

- Maintaining the confidentiality of your information when participating in research is critical. We will take specific steps to protect your personal information.

- Your name will be used only to return test results to you and will not be used for any other purpose.

- Because we will use the barcode system to manage, your name is not displayed anywhere. We do not know which patient's name is on the sample once a barcode is attached to it.

- Your personal information and any information you provide for this research will be kept secret and used for research purposes only.

- If the research results are published, we will not publish your name or any other personal information.

- We will keep your remaining samples and related information for five years after the study is completed.

Who to Contact?

If you have any questions or concerns related to this research topic, you can contact:

- Principle investigator: Assoc. Prof. Bui Thi Viet Ha- Vietnam National Children's Hospital, VNU University of Science, 334 Nguyen Trai, Thanh Xuan, Hanoi, Mobile phone 0906269956;

- Investigator (back up) : Dr. Dang Thi Hong Thang - Hanoi Obstetrics & Gynecology Hospital No. 929 La Thanh, Dong Da, Hanoi, Mobile phone 0979213268

You can also contact the ethics committee of Dinh Thien Hoang Institute of Medicine if you have questions about your rights if participating in research. Contact information for the Ethics Committee is provided below.

Hoa Lac Hi-Tech Park, Km 29 Thang Long high-way, Hanoi, Tel: +84 2439 913 177

Part II: Certificate of Consent

This consent form is designed to check that you understand the purpose of the study, that you are aware of your rights as a participant and to confirm that you are willing to take part.

Please tick as appropriate		
	YES	NO
1. I have read the leaflet describing the study (PART I)		
2. I have received sufficient information about the study for me to decide whether to take part.		
3. I understand that I am free to refuse to take part if I wish		
4. I understand that I may withdraw from the study at any time without having to provide reason.		
5. I know that I can ask for further information about the study from the research team.		
6. I understand that all information arising from the study will be treated as confidential.		
7. I know that it will not be possible to identify any individual respondent in the study report, including myself.		
8. I agree to take part in the study.		
I confirm that quotations from the interview can be used in the final research report and other publications. I understand that these will be used anonymously and that no individual respondent will be identified in such a report.		
Signature: Date:		
Name in block letters, please:		
